# Advances from the Office of Naval Research STEM Grand Challenge: expanding the boundaries of intelligent tutoring systems

**DOI:** 10.1186/s40594-018-0111-x

**Published:** 2018-04-12

**Authors:** Scotty D. Craig, Arthur C. Graesser, Ray S. Perez

**Affiliations:** 1 Human Systems Engineering, Arizona State University, 7271 E Sonoran Arroyo Mall, Santa Catalina Hall, Ste. 150, Mesa, 85212 AZ USA; 2 Institute for Intelligent Systems, University of Memphis, Memphis, USA; 3 Warfighter Performance Department, Office of Naval Research, Arlington, USA

**Keywords:** Intelligent tutoring systems, ITS integration, Office of Naval Research, Personalized learning

## Abstract

This special issue presents evaluations of four intelligent tutoring systems. These systems were funded under the Office of Naval Research’s STEM Grand Challenge for intelligent tutoring systems. The systems each represent aspects of how ITS can address STEM education or how aspects of multiple systems can be integrated to support STEM education. The selected papers also provide empirical evidence for the effectiveness of each system. The current paper provides an overview of the Office of Naval Research STEM Grand Challenge program, the systems funded under the program, and summaries of the articles within this special issue.

The Office of Naval Research initiated a program of research to develop intelligent tutoring technologies in response to then President Obama’s three overarching priorities for Science Technology Engineering Mathematics (STEM) education. These priorities were to (1) increase STEM literacy, (2) improve the quality of math education and science teaching, and (3) expand STEM education and career opportunities. This program was also to address the Chief Naval Officer of the Navy’s goal to grow top technical talent scientists and engineers who will lead tomorrow’s Navy by addressing the aging work force and increasing the candidate pool of scientist and engineers that would work in Navy laboratories. This initiative made sense in light of the research by the Organization for Economic, Cooperation and Development that reported that a gain in math and science performance could significantly increase a nation’s gross domestic product (OECD 2013).

The challenge’s goal was to develop intelligent tutor technologies for middle/high schools within the STEM education area that improve student retention, reasoning, and problem solving by at least two standard deviations. One salient benefit would be that the resultant technologies could be easily modified to support Navy training. However, the fundamental long-term goal was to develop “*better*, *faster*, *and cheaper*” intelligent tutors.

## Funded projects

ONR released a request for proposals for phase 1, in which four awards would be made of up to $1.5 million each. At the conclusion of phase 1, ONR would select two awards for military applications of up to $1 million each. The four awards received in phase 1 were:

1. *SKOPE-IT*, University of Memphis, with developers Xiangen Hu and Arthur Graesser. *SKOPE-IT* (Shareable Knowledge Objects as Portable Intelligent Tutors) is the integration of two ITS technologies: *AutoTutor* and *ALEKS*. *AutoTutor* is an intelligent tutoring system that holds conversations with learners in natural language and closely models expert human tutors within its implemented dialogue framework (Graesser 2016). The tutor has produced learning gains across several domains, including computer literacy, physics, critical thinking, and mass casualty training. Overall, research shows that Auto-Tutor has produced learning gains that are on average about 0.8 standard deviation units above students in control conditions who read static instructional materials from textbooks for an equivalent amount of time. *ALEKS* (Assessment and Learning Knowledge Spaces) is a web-based intelligent learning and assessment system that teaches mathematics for K-12 (Falmagne et al. 2013). For the *SKOPE-IT* project, AutoTutor has been extended to discuss problems presented in *ALEKS* for algebra. By leveraging the expertly produced math problems and worked-examples from *ALEKS*, the combined system provides students interactive mini-dialogues that focus on math reading comprehension that includes the concepts behind the steps required to solve Algebra problems.
2. *High school AP Physics tutor* Raytheon BBN Technologies, with developers Rohit Kumar and Matt Roy. The *BBN Learning Platform* (*Learnform*) is a domain-independent, problem-solving-based, online learning platform (Kumar et al. 2015). Students solve problems at their own pace or request help which guides them through the steps of a problem. Teachers can view automated reports through a dashboard. *Learnform* includes a web-based workbench for authoring and publishing new content.
3. *MathSpring*, University of Massachusetts-Amherst, with developers Beverly Woolf, Christina Heffernan, Neil Heffernan, Joseph Beck, and Ivon Arroyo. *MathSpring* is a math practice environment inside an adaptive tutor that provides math instruction embedded within extensive hints, help, and links to external instructional videos (Arroyo et al. 2014). The system is an integration of Wayang Outpost and ASSISTments. Wayang Outpost (Beal et al. 2007) is an online tutoring system for high school level mathematics achievement that focuses on SAT math skills. ASSISTments is a platform that is used by teachers to assign digital homework and classroom activities (Heffernan and Heffernan 2014). It comes with a library of math content but is also flexible so teachers can put in any questions they want. The system has been used by 50,000 students per year and has been shown to help improve student learning by 75%. The combined system, MathSpring, had tens of thousands of student users during the evaluation period and produced nearly a letter grade improvement in students.
4. *Dragoon*, Arizona State University, with developers Dr Kurt Van Lehn and John Wetzel. *Dragoon* is an intelligent tutoring system that coaches students as they learn how to construct mental models of dynamic complex systems (VanLehn et al. 2016). Modeling is one of seven fundamental practices in both the Common Core State Standards for Mathematics and the Next Generation Science Standards. It is also important in many university-level science and engineering classes. *Dragoon* has been in continuous use in two ASU courses for several years. It has been used in high school biology, physics, chemistry, and earth science classes. In a high school physiology class, *Dragoon* students learned significantly more than students studying the same content using only paper-and-pencil exercises. *Dragoon* allows students to construct sophisticated models of time-varying systems (e.g., ecosystems, projectile dynamics, blood glucose regulation) by just drawing node-link diagrams, while receiving feedback and hints from Dragoon.

The features of these systems include the standard components of all ITS, namely a set of subject matter knowledge components, a model of the students’ knowledge states, intelligent pedagogical strategies, and an interface for learner-computer interaction (Woolf 2010). However, these systems typically had significant advances over the traditional ITS on one or more of these components. Some systems had pedagogical strategies that mirror interactions between expert teachers and individual students. Some had an interface with conversational computer agents. Some had automated knowledge elicitation technologies for developing instructional content. And others had a science-based understanding of how students in different age groups learn.

Phase 2 of the ONR effort on building ITS consisted of two Navy training applications. The first is the development of ElectronixTutor to help sailors learn about electronic circuits. University of Memphis (see Graesser et al. 2018, current issue) led this effort in an ITS that integrated components from all four of the teams in phase 1 of the ONR STEM Challenge. This was a unique effort that attempts to deliver the right learning resource for the right sailor at the right time, based on the sailor’s prior performance history. The second is an intelligent tutor for robotic surgery, led by Rohit Kumar and David Diller at Raytheon/BBN Technologies (see Skinner et al. 2018, current issue). Both of these tutors are currently under development and in the process of being tested.

## Article summaries

This special issue provides two example articles from the phase 1 projects and two articles from the phase 2 projects. These articles provide an overview and snapshot of the advanced ITS that have recently been supported by ONR. ONR has played a major role in leading the development of these systems throughout the history of ITS and before these systems were supported by National Science Foundation, Institute of Education Sciences, and research foundations (Chipman 2015; Sabo et al. 2013). The four primary articles are followed by a commentary by Fletcher 2018 which reflects on these developments as well as the state of ITS in general and assessments of these systems on learning gains (see also Kulik and Fletcher 2015).

The first article is *SKOPE-IT* (*Shareable Knowledge Objects as Portable Intelligent Tutors*)*: Overlaying Natural Language Tutoring on an Adaptive Learning System for Mathematics* by Nye et al. (2018). *SKOPE-IT* is a natural language tutoring system on mathematics. This system is a hybrid of two highly effective tutoring systems. The first system, *ALEKS* (Assessment and Learning in Knowledge Spaces), is a mastery-based tutoring system based off of Knowledge Space Theory (Craig et al. 2013; Falmagne et al. 2013; Hu et al. 2012). To produce SKOPE-IT, the ALEKS architecture was combined with the AutoTutor Conversation Engine (Graesser 2016). This version of AutoTutor is a web service that allows for natural language conversations with one or more conversational agents. The article describes an evaluation comparing the SKOPE-IT system versus the ALEKS system with college-level algebra students.

The second article by Inventado et al. (2018), *Contextual Factors Affecting Hint Utility*, describes the use of a new hint utility within a mathematics-based tutoring system, *ASSISTments*. It describes a randomized trial of the system with the hint utility. The article provides evidence for the impact of the hint utility, a case study of how randomized trials can be implemented with *ASSISTments*, and lessons learned from the implementation.

The third article by Skinner, Diller, Cannon-Bowers, Smith, Tanaka, Julian, Kumar, and Perez, *Development and Application of a Multi-Modal Task Analysis to Support Intelligent Tutoring of Complex Skills* (Skinner et al. 2018), describes the development of an Intelligent Tutoring System for training Robotic Assisted Laparoscopic Surgery (RALS). This article provides a strong example for the potential that increasing the consideration of the end user, usability, and human factors/human systems can have for improving educational technology (Roscoe et al. 2017). The argument is made for the use of cognitive task analysis (CTA) methods for gathering reliable training corpora. However, the article also argues for the need for a new type of CTA called multi-modal task analysis (MMTA) that elicits knowledge for cognitive, psychomotor, and perceptual skills from experts. The article provides an example of this new technique and how it was used within the RALS training tasks that model the RALS skills.

The fourth article by Graesser et al. (2018), *ElectronixTutor: An intelligent tutoring system with multiple learning resources for electronics*, was a monumental contribution by 25 authors from academia and industry across eight different institutions. The *ElectronixTutor* plays off the success and lessons learned from the SKOPE-IT project (Nye et al. 2018) to create an intelligent learning resource that integrates elements from five highly successful intelligent Tutoring Systems (AutoTutor, Dragoon, LearnForm, ASSISTments, BEETLE-II) as well as traditional reading of text materials. The article is both a review paper and an example case for system integration (i.e., ElectronixTutor). The article provides a summary of research for each component system and best practices for ITS development. The article also provides an example case for system integration within the educational technology area. It provides a wealth of history and lessons learned from a wide section of the Intelligent Tutoring System Literature.
